# Association of the lymphocyte-to-monocyte ratio, mean diameter of coronary arteries, and uric acid level with coronary slow flow in isolated coronary artery ectasia

**DOI:** 10.1186/s12872-021-01952-4

**Published:** 2021-03-30

**Authors:** Zhuoxuan Yang, Jiansong Yuan, JinGang Cui, Hao Guan, Shubin Qiao

**Affiliations:** Department of Cardiology, Chinese Academy of Medical Sciences Fuwai Hospital, No. 167 North Lishi Road, Xicheng District, Beijing, 100037 China

**Keywords:** Isolated coronary artery ectasia, Coronary slow flow, Uric acid, Mean diameter of arteries, Lymphocyte-to-monocyte ratio

## Abstract

**Background:**

The pathophysiology of isolated coronary artery ectasia (CAE) with the coronary slow flow (CSF) phenomenon is still unclear. The purpose of this study was to investigate the risk factors for isolated CAE complicated with CSF.

**Methods:**

A total of 126 patients with isolated CAE were selected retrospectively. The patients were grouped into the no CSF (NCSF) group (n = 55) and the CSF group (n = 71) according to the corrected thrombolysis in myocardial infarction (TIMI) frame count (CTFC). Data on demographics, laboratory measurements, left ventricular ejection fraction (LVEF), left ventricular end-diastolic diameter (LVEDd), CTFC and diameters of three coronary arteries were collected.

**Results:**

The proportions of males (84.5% vs. 61.8%, *p* = 0.004) and patients with a smoking history (63.4% vs. 43.6%, *p* = 0.021) were higher in the CSF group than in the NCSF group. The neutrophil-to-lymphocyte ratio (NLR) (2.08(1.68–3.21) vs. 1.89 ± 0.58, *p* = 0.001), mean diameter of coronary arteries (mean D) (5.50 ± 0.85 vs. 5.18 ± 0.91, *p* < 0.001), and uric acid (URIC) level (370.78 ± 109.79 vs. 329.15 ± 79.71, *p* = 0.019) were significantly higher in the CSF group, while the lymphocyte-to-monocyte ratio (LMR) (4.81 ± 1.66 vs. 5.96 ± 1.75, *p* < 0.001) and albumin (ALB) level (44.13 ± 4.10 vs. 45.69 ± 4.11, *p* = 0.036) were lower. Multivariable logistic analysis showed that the LMR (odds ratio: 0.614, 95% CI: 0.464–0.814, *p* = 0.001), mean D (odds ratio: 2.643, 95% CI: 1.54–4.51, p < 0.001) and URIC level (odds ratio: 1.006, 95% CI: 1.001–1.012, *p* = 0.018) were independent predictors of CSF in CAE.

**Conclusions:**

The LMR was a negative independent predictor of CSF in isolated CAE, while URIC level and mean D were positive independent predictors.

## Background

Isolated coronary artery ectasia (CAE) refers to the local diameter of a coronary artery being dilated more than 1.5 times the adjacent normal vessels and having no obvious obstruction [1–3], with a incidence range between 0.1%-0.32% in coronary angiography [4]. The coronary slow flow (CSF) phenomenon is coronary angiography that shows no obvious stenosis but slow forward flow perfusion [5,6]. The incidence of the CSF phenomenon in coronary angiography is less than 1% [6]. However, it can cause serious cardiac events, such as angina pectoris, myocardial infarction, malignant arrhythmia and even sudden death [7].

There is no consensus on the physiological mechanism of the above two diseases. Although a change in the vascular diameter will affect the velocity of flow, not all patients with CAE have CSF. Since the morbidity of these two diseases is low, large sample studies are lacking. Some small sample studies have suggested that the lymphocyte-to-monocyte ratio (LMR), neutrophil-to-lymphocyte ratio (NLR), albumin (ALB) level, high-sensitivity C-reactive protein (hs-CRP) level and uric acid (URIC) level are risk factors for CSF or CAE [1–3,6,8–12]. Therefore, the aim of this study was to investigate the risk factors for CSF in patients with isolated CAE.

## Materials and methods

### Subjects

From January 2010 to December 2019, 541 patients who underwent coronary angiography in our hospital and were diagnosed with isolated CAE were selected. The inclusion criteria were as follows: the diameter of the local coronary artery was more than 1.5 times larger than that of the adjacent normal segment with a degree of stenosis < 20%, and cardiac ultrasound showed that the heart structure and left ventricular ejection function (LVEF) were normal. The exclusion criteria were as follows: acute coronary syndrome, coronary spasm, coronary artery bypass graft, valve disease, congenital heart disease, Kawasaki disease, left ventricular or ventricular septal hypertrophy, immunological disease, malignant tumour, severe cerebrovascular disease, severe hepatic or renal insufficiency (creatinine > 132 µmol/L, AST or ALT > 2 times the upper level of normal), haematological system disease or haemoglobin < 90 g/L, and received steroid hormone treatment or acute inflammatory disease within 1 month. A total of 126 patients with isolated CAE were included and grouped into the CSF group (n = 71) and the no CSF (NCSF) group (n = 55).

### Angiography data and frame counting

The methods used to assess coronary flow included the thrombolysis in myocardial infarction (TIMI) grade and the corrected TIMI frame count (CTFC). The TIMI grade is divided into four grades(Grade 0 = No perfusion, Grade 1 = Penetration without perfusion,Grade 2 = Partial perfusion,Grade 3 = Complete perfusion).The CTFC method was first described by Gibson et al. [13]. The frames were collected at a rate of 30 frames/s, and the number of frames from the start to the distal coronary artery was counted. In the first frame, the contrast agent completely fills the coronary artery, and the forward motion of the contrast agent can be observed. In the final frame, the contrast agent reaches a certain landmark of the coronary artery. The sign of the left anterior descending (LAD) artery is the "whale tail" or "hay fork" at the distal bifurcation. The landmark of the left circumflex (LCX) artery is the distal bifurcation of the obtuse marginal branch, and the first branch of the posterolateral artery is used for the right coronary artery (RCA). Because the LAD artery is longer than the LCX artery, the TIMI frame count (TFC) for this artery should be divided by 1.7 to obtain the CTFC. The average CTFC (mean CTFC) was obtained by summing the CTFC of 3 arteries and then dividing by 3. Gibson found that 95% CI normal flow was ≥ 15 to ≤ 27 frames [13], therefore we used the CTFC ≥ 27 frames as a cutoff of coronary slow flow. This method is more objective and has been more widely adopted in other studies [5,11,14].

### Demographics and laboratory measurements

Age, sex, body mass index (BMI), history of hypertension, type 2 diabetes mellitus, medication, smoking and drinking, white blood cell count, neutrophil count, lymphocyte count, monocyte count, red blood cell count, haemoglobin count, blood cell specific volume, red blood cell distribution width, mean platelet volume (MPV), mean red blood cell volume, C reactive protein (CRP), high-sensitivity C reactive protein (hs-CRP), ALB, URIC, creatinine, total bilirubin (TBIL), indirect bilirubin (IBIL), triglycerides (TG), total cholesterol (TC), high-density lipoprotein cholesterol (HDL-C), low-density lipoprotein cholesterol (LDL-C), glycated haemoglobin (HbA1C), NT-proBNP, LVEF, and left ventricular end-diastolic diameter (LVEDd) were noted. The CTFC and vascular diameter were also assessed by two interventional physicians.

Written informed consent was obtained from each patient included in this study. The study protocol conformed to the ethical guidelines of the 1975 Declaration of Helsinki and was approved by the institution's ethics committee on research on humans.

### Statistical analysis

All analyses were performed using the Statistical Package for the Social Sciences version 22.0 (IBM Corp., Armonk, New York, USA) and MedCalc software (bvba 19, Ostend, Belgium). Continuous variables are presented as the means ± standard deviations (normal distribution) or as the medians (non-normal distribution). Categorical variables are presented as absolute values and percentages. Comparisons of categorical variables between the two groups were performed using the chi-squared (χ2) test. The Kolmogorov–Smirnov test was performed to assess whether the variables were normally distributed. Student's t-test or the Mann–Whitney U test was used to compare the continuous variables between the two groups according to whether they were normally distributed. Spearman's rho correlation analysis was performed to describe the degree of correlation between the parameters related to the mean TFC. To identify the independent predictors of CSF, univariate and multivariate logistic regression analyses (backward LR method) were performed. The variables with P < 0.1 in the univariate analysis were incorporated into the multivariate logistic regression analysis. Receiver operating characteristic (ROC) curve analysis was performed to determine additional assets of parameters found to be independent predictors of CSF. The optimal cut-off value was calculated from the point of maximum sensitivity and specificity (Youden's index). A P < 0.05 was considered as statistically significant.

## Results

### Baseline characteristics

A comparison of the demographic and clinical characteristics is shown in Table 1. The proportions of males (84.5% vs. 61.8%, *p* = 0.004) and patients with a smoking history (63.4% vs. 43.6%, *p* = 0.021) were significantly higher in the CSF group than in the NCSF group.The number of CAE cases in the LAD and LCX arteries was significantly higher in the CSF group; however, there was no statistically significant difference in terms of the number of CAE cases in the RCA between groups. The number of CAE in single vessel was higher in the NCSF group. However, the number of CAE in multiple vessels was higher in CSF than NCSF group.

**Table 1:** Demographic and clinical characteristics of the study population

	NCSF group (n = 55)	CSF group (n = 71)	*p*
Age	55.73 ± 11.93	55.85 ± 11.06	0.954
Male	34 (61.8%)	60 (84.5%)	0.004
BMI	26.29 ± 4.97	27.15 ± 3.41	0.251
Smoking history	24 (43.6%)	45 (63.4%)	0.021
Anti-platelet drugs	30 (54.5%)	36 (50.7%)	0.402
β-blockers	19 (34.5%)	23 (32.4%)	0.474
ACE inhibitors	6 (10.9%)	5 (7%)	0.326
ARBs	8 (14.5%)	10 (14.1%)	0.569
Statins	26 (47.3%)	31 (43.7%)	0.411
Hypertension	36 (65.5%)	42 (59.2%)	0.296
Diabetes	13 (23.6%)	10 (14.1%)	0.276
LVEF	64.11 ± 4.20	48.68 ± 4.16	0.264
LVEDd	48.44 ± 4.33	48.68 ± 4.16	0.753
CAE in LAD (%)	29 (52.7%)	52 (73.2%)	0.044
CAE in LCX (%)	14 (25.5%)	41 (57.7%)	0.001
CAE in RCA (%)	31 (56.4%)	52 (73.2%)	0.111
CAE in 1 vessel	33 (60%)	21 (29.6%)	0.001
CAE in 2 vessels	15 (27.3%)	26 (36.6%)	0.001
CAE in 3 vessels	7 (12.7%)	24 (33.8%)	0.001

A comparison of the laboratory variables between the two groups is shown in Table 2. The differences in the neutrophil-to-platelet ratio (NPR), TG, TC, HDL-C, LDL-C, HbA1C, LVEDd, LVEF, NT-proBNP and estimated glomerular filtration rate (eGFR) were not significant between the groups. The diameters of the LAD artery (*p* = 0.002), LCX artery (*p* = 0.001) and mean D (*p* < 0.001) were significantly larger in the CSF group; however, the diameter of the RCA was not significantly different between the groups. The LAD-CTFC(*p* < 0.001), LCX-CTFC(*p* < 0.001), RCA-CTFC(*p* < 0.001), Mean CTFC (*p* < 0.001), NLR (*p* = 0.001) and URIC level (*p* = 0.019) were higher in the CSF group, while the LMR (*p* < 0.001) and ALB level (*p* = 0.036) were significantly lower in the CSF group.

**Table 2:** Comparison of laboratory variables in the study population

	NCSF group (n = 55)	CSF group (n = 71)	*p*
LAD diameter (mm)	5.52 ± 1.33	6.27 ± 1.25	0.002
LCX diameter (mm)	5.05 ± 1.45	5.91 ± 1.39	0.001
RCA diameter (mm)	5.91 ± 1.55	6.37 ± 2.02	0.173
Mean D	5.18 ± 0.91	5.50 ± 0.85	< 0.001
LAD CTFC	20 (15–24)	29 (22.75–34)	< 0.001
LCX CTFC	21 (17–24)	32 (25.75–38)	< 0.001
RCA CTFC	19 (14–23)	29 (23.5–35.25)	< 0.001
Mean CTFC	19.29 (17.45–23.06)	29.55 (24.7–34.05)	< 0.001
WBC	6.36 ± 1.61	6.60 ± 1.58	0.399
Neutrophils	3.75 ± 1.15	4.11 ± 1.22	0.091
Lymphocytes	2.08 ± 0.60	1.88 ± 0.69	0.08
Monocytes	0.37 ± 0.11	0.41 ± 0.13	0.064
NLR	1.89 ± 0.58	2.08 (1.68–3.21)	0.001
NPR	0.0088 ± 0.0033	0.030 ± 0.157	0.317
LMR	5.96 ± 1.75	4.81 ± 1.66	< 0.001
hsCRP	1.53 ± 1.67	1.98 ± 2.26	0.218
CRP	2.65 ± 2.16	3.33 ± 3.34	0.192
HGB	147.82 ± 14.46	152.15 ± 14.48	0.098
ALB	45.69 ± 4.11	44.13 ± 4.10	0.036
URIC	329.15 ± 79.71	370.78 ± 109.79	0.019
HbA1C	6.27 ± 1.21	6.12 ± 1.08	0.469
Fasting Glucose	6.332.13	5.79 ± 2.03	0.152
TG	1.81 ± 1.12	1.93 ± 1.59	0.653
TC	4.46 ± 1.08	4.50 ± 1.16	0.820
HDL-C	1.18 ± 0.34	1.20 ± 0.38	0.704
LDL-C	2.74 ± 0.91	2.74 ± 0.95	0.970
eGFR	103.65 ± 32.28	102.09 ± 25.30	0.762
NT-proBNP	114.53 ± 143.88	142.57 ± 196.85	0.419

NPR:Neutrophil to Pletelets Ratio; Mean D: Mean diameter of LAD, LCX and RCA; NLR: Neutrophils to Lymphocytes Ratio; LMR:Lymphocytes to Monocytes Ratio; CRP:C-reactive protein; ALB: albumin

### Predictors of CSF

In the Spearman correlation analyses (Table 3), the LMR (*r* = -−0.21, *p* = 0.026), ALB level (*r* = -−0.187, *p* = 0.036) and male sex (*r* = -−0.265, *p* = 0.003) were negatively correlated with CSF, whereas the diameters of the LAD artery (*r* = 0.297, *p* < 0.001), LCX artery (*r* = 0.218, *p* = 0.016) and RCA (*r* = 0.235, *p* = 0.01), as well as the mean diameter of coronary arteries (r = 0.337, *p* < 0.001), NLR (*r* = 0.245, *p* = 0.009) and URIC level (*r* = 0.218, *p* = 0.021), were positively correlated with CSF. Smoking history (*r* = 0.2, *p* = 0.026) was also positively correlated with CSF.

**Table 3:** Spearman’s rho correlation analysis between the CSF with risk factors

Variable	r	*p*
Gender	− 0.265	0.003
Smoking history	0.2	0.026
LAD diameter (mm)	0.297	< 0.001
LCX diameter (mm)	0.218	0.016
RCA diameter (mm)	0.235	0.01
Mean D (mm)	0.337	< 0.001
NLR	0.245	0.009
LMR	− 0.21	0.026
URIC	0.218	0.021
ALB	− 0.187	0.036

To further explore the independent predictor(s) of CSF, univariable and multivariable logistic regression model analyses were performed based on the correlation analysis results (Table 4). We performed multivariate analysis by using the backward LR method. A decreased LMR (OR: 0.614; 95% CI: 0.464–0.814; *p* = 0.001), an increased mean D (OR: 2.634; 95% CI: 1.54–4.51; *p* < 0.001) and the URIC level (OR: 1.006; 95% CI: 1.001–1.012; *p* = 0.018) were found to be independent predictors of CSF development.

**Table 4:** Univariable and multivariable logistic regression analyses on the presence of slow coronary flow

Variables	Univariable		Multivariable	
	OR (95% CI)	*p*	OR (95% CI)	*p*
Male	3.369 (1.451–7.820)	0.005	1.601 (0.443–5.781)	0.437
Smoking history	2.236 (0.218–0.918)	0.028	1.478 (0.505–4.324)	0.476
LAD diameter	1.584 (1.169–2.147)	0.003		
LCX diameter	1.531 (1.169–2.005)	0.002		
Mean diameter of coronary arteries	2.62 (1.543–4.117)	< 0.001	2.634 (1.54–4.51)	< 0.001
URIC	1.005 (1.001–1.009)	0.023	1.006 (1.001–1.012)	0.018
NLR	2.18 (1.321–3.599)	0.002	1.741 (0.854–3.549)	0.127
LMR	0.67 (0.530–0.847)	0.001	0.614 (0.464–0.814)	0.001
ALB	0.911 (0.833–0.995)	0.039	0.917 (0.826–1.019)	0.107

OR: odds ratio

**Fig. 1:**
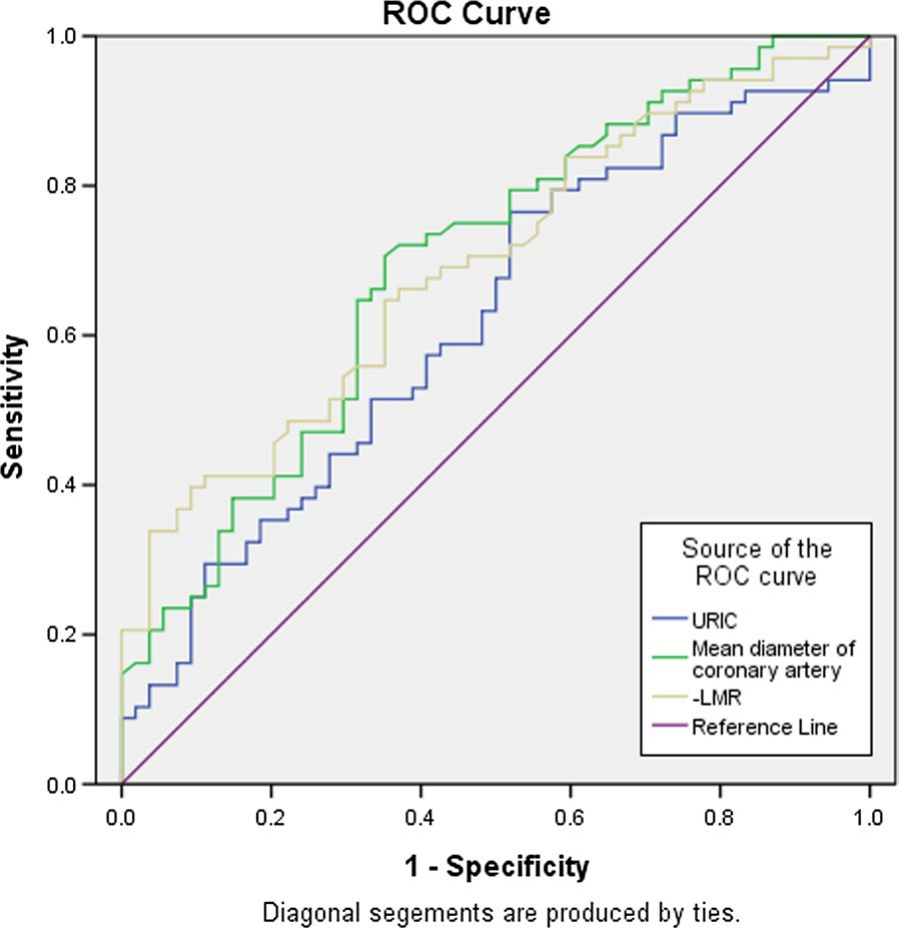
Receiver operating characteristic (ROC) curve of Mean D, LMR and URIC

ROC curve analysis demonstrated that the specificity of the mean D > 5.76 mm in predicting CSF was 70.59%, and the sensitivity was 64.81% (AUC = 0.696, *p* < 0.001). It was revealed that using a cut-off level of an LMR < 4.11 predicted CSF with a sensitivity of 90.9% and a specificity of 40.85% (AUC = 0.698, *p* < 0.001). The URIC level was found to have an AUC of 0.616 (*p* = 0.026) with an optimal URIC cut-off value of 314.39 μmol/L (sensitivity 74.65%, specificity 49.09%) (Fig. 1).

## Discussion

The main discoveries of this study can be summarized as follows: 1) the inflammatory indicators were higher in the CSF group than in the NCSF group; 2) The LMR was a negetive independent predictor of CSF in isolated CAE, while URIC level and mean D were positive independent predictors.

### Patho-immunologic background

At present, the exact pathogenesis of CAE and CSF is not clear. Hypotheses suggest that chronic inflammation, atherosclerosis, endothelial dysfunction and oxidative stress may participate in the pathological processes of the two diseases [1–12,15]. Many studies have focused on inflammation, and few have investigated other possible causes.

Monocytes and lymphocytes are vital immune cells. They also play an important role in the process of atherosclerosis [6,9,10,16–21]. In response to the stimulation of inflammatory cytokines, monocytes are recruited to the intima and subintima via the assistance of adhesion molecules during the initial stage [6,18]. After migration, monocytes differentiate into macrophages, devouring oxidized LDL-C and releasing a large number of inflammatory factors, such as interleukin (IL)-1, IL-6, tumour necrosis factor (TNF)-α, and macrophage colony-stimulating factor, which attract more monocytes [21]. Lymphocytes can enhance the immune response by regulating catecholamine and cortisol levels in the anti-inflammatory milieu. However, as catecholamine and cortisol levels increase, the lymphocyte numbers will gradually decline. The hypothesis concerning this debatable condition includes the downregulation of lymphocyte differentiation and proliferation, the upregulation of lymphocyte apoptosis and redistribution within the lymphoid organs [17,18,21].

### Role of LMR and URIC

Because the LMR combines two kinds of immune cells, it has long been used as an indicator of the condition and treatment of cancer patients [22,23]. In recent years, studies have shown that the LMR is related to stable angina [17,18], acute coronary syndrome (ACS)^[[[[24]]]]^ and CSF [6], and it can even be used as an independent predictor of major adverse cardiac events (MACEs) [20,21,24]. Yildirim et al. [16]. found that the expression of active markers on monocyte-derived dendritic cells in patients with CAE was significantly higher than that in patients with coronary artery disease (CAD). Yayla [6] investigated the LMR in normal coronary arteries with and without CSF and found that a low LMR was independently associated with the CSF phenomenon. Kalyoncuoglu et al. [9]. found that a high monocyte-to-HDL-C ratio (MHR) and low LMR were independent predictors of slow flow/no reflow in patients with non-ST-elevated myocardial infarction (NSTEMI). In our study, the LMR was significantly lower in the CSF group and was found to be an independent risk factor for CSF development in isolated CAE.

In previous studies, elevated URIC levels were associated with an activated inflammatory response in patients with STEMI and associated with poor short-term and long-term outcomes [25–27]. Mandurino-Mirizzi [26] found that in patients with STEMI, an elevated serum URIC level was related to a large infarct size, which might be explained by impaired myocardial reperfusion. In our study, the URIC level was an independent predictor of CSF in isolated CAE, consistent with the findings of Mandurino-Mirizzi [26]. Moreover, URIC can impair nitric oxide generation in endothelial cells through multiple pathways, leading to endothelial dysfunction [3,12,28,29]. It can also induce vascular smooth muscle cell (VSMC) proliferation and differentiation by activating mitogen-activated protein kinases [30]. VSMCs migrate from the middle layer to the subintima and participate in the formation and development of atherosclerotic plaques.

Both the LMR and URIC level play an exclusive role in inflammation and the early formation of atherosclerotic plaques and growth of the lipid core, suggesting that early severe inflammation and rapid atherosclerotic development in CAE patients might be an important pathogenesis for the CSF phenomenon.

### Role of mean D

The deep impression of CAE in angiography is local or diffuse vascular expansion. It is viewed in the cross-section of a coronary artery as circular according to the flow equation Q = πr^2^^v, where Q is traffic, r is the radius, and v is flow velocity. It is known that when the traffic is constant, flow velocity is inversely proportional to the square of the radius. This was consistent with our study, showing that the mean D in the CSF group was larger than that in the NCSF group.

### Potential predictors of CSF in CAE

Somaschini [31] found that the NPR, a new promising inflammatory biomarker, was an independent predictor of short-term mortality in STEMI patients who underwent primary percutaneous coronary intervention (PCI). Yilmaz [11] suggested that the NLR was significantly higher in patients with CAE, CSF and CAD than in normal subjects and was an independent predictor of these diseases. Cetin [8] found that the ALB level was significantly lower in the CSF group than in the normal group and was an independent predictor of CSF. In our study, however, the difference in the NPR between groups was not significant. The NPR is increased significantly in acute inflammatory and thrombotic or haemorrhagic disease [31–33]. However, the thrombotic or haemorrhagic phenomenon is absent in coronary artery ectasia with or without slow flow. The NLR and ALB level were statistically significant in both groups but did not have significant predictive power. A possible reason might be the different control groups used in these studies. Yilmaz [11] and Cetin [8] studied normal people with no obvious inflammation as controls. However, the control group in our study included CAE patients who had an elevated level of inflammatory biomarkers compared to normal subjects; therefore, the prediction ability of the inflammatory indicators did not reach statistical significance.

## Limitations

There were some limitations to this study. The first limitation relates to its retrospective design. Some risk factors, such as the matrix metalloproteinase family, tissue inhibitors of metalloproteinases [34,35] and adropin [2], which play a significant role in CAE development, could not be examined. Second, multivariable logistic regression analyses were performed to identify independent predictors of CSF in CAE; however, it was impossible to control for unknown confounders. In addition, this study reviewed the cases in one centre over a 10-year period, and due to the low morbidity of both diseases, the sample size was still not large enough to reflect more latent risk factors. More studies are needed to investigate the pathogenesis of CAE in patients with CSF.

## Conclusion

Patients with isolated CAE combined with CSF have a high URIC level and a large diameter of coronary arteries and a low LMR. This study revealed that severe inflammatory reactions and atherosclerosis development might be targets for future pathogenesis studies in patients with isolated CAE combined with CSF.

## Data Availability

The datasets used and analysed during the current study are available from the corresponding author on reasonable request.

## Abbreviations

LMR: Uymphocyte-to-monocyte ratio.
CSF: Uoronary slow flow.
CAE: Uoronary artery ectasia
Mean D: Mean diameter of coronary arteries
URIC: Uric acid
NPR: Neutrophil to platelet Ratio
